# Improving Outcomes in Infants of HIV-Infected Women in a Developing Country Setting

**DOI:** 10.1371/journal.pone.0003723

**Published:** 2008-11-14

**Authors:** Francine Noel, Sapna Mehta, Yuwei Zhu, Patricia De Matteis Rouzier, Abdias Marcelin, Jian R. Shi, Claudine Nolte, Linda Severe, Marie Marcelle Deschamps, Daniel W. Fitzgerald, Warren D. Johnson, Peter F. Wright, Jean W. Pape

**Affiliations:** 1 Les Centres GHESKIO, Port-au-Prince, Haiti; 2 Department of Pediatrics, Division of Infectious Diseases, Vanderbilt University, School of Medicine, Nashville, Tennessee, United States of America; 3 Department of Biostatistics, Vanderbilt University, Nashville, Tennessee, United States of America; 4 Department of Medicine, Division of Infectious Diseases and International Health, Weill Medical College of Cornell University, New York, New York, United States of America; 5 Division of Infectious Disease and International Medicine, Dartmouth Medical School, Lebanon, New Hampshire, United States of America; Tulane University, United States of America

## Abstract

**Background:**

Since 1999 GHESKIO, a large voluntary counseling and HIV testing center in Port-au-Prince, Haiti, has had an ongoing collaboration with the Haitian Ministry of Health to reduce the rate of mother to child HIV transmission. There are limited data on the ability to administer complex regimens for reducing mother to child transmission and on risk factors for continued transmission and infant mortality within programmatic settings in developing countries.

**Methods and Findings:**

We analyzed data from 551 infants born to HIV-infected mothers seen at GHESKIO, between 1999 and 2005. HIV-infected mothers and their infants were given “short-course” monotherapy with antiretrovirals for prophylaxis; and, since 2003, highly active antiretroviral therapy (HAART) when clinical or laboratory indications were met. Infected women seen in the pre-treatment era had 27% transmission rates, falling to 10% in this cohort of 551 infants, and to only 1.9% in infants of women on HAART. Mortality rate after HAART introduction (0.12 per year of follow-up [0.08–0.16]) was significantly lower than the period before the availability of such therapy (0.23 [0.16–0.30], P<0.0001). The effects of maternal health, infant feeding, completeness of prophylaxis, and birth weight on mortality and transmission were determined using univariate and multivariate analysis. Infant HIV-1 infection and low birth weight were associated with infant mortality in less than 15 month olds in multivariate analysis.

**Conclusions:**

Our findings demonstrate success in prevention of mother-to-child HIV transmission and mortality in a highly resource constrained setting. Elements contributing to programmatic success include provision of HAART in the context of a comprehensive program with pre and postnatal care for both mother and infant.

## Introduction

In 2007, 420,000 human immunodeficiency virus type 1 (HIV) infections are estimated to have occurred in children as a result of mother to child transmission (MTCT) during pregnancy (intra-uterine), during birth (intra-partum) or from breastfeeding [1]. The vast majority of such infections occurred in low and middle- income countries [1].

In high income countries, MTCT is now rare (<2%) due to universal use of highly active antiretroviral therapy (HAART) for pregnant women, elective caesarean sections and avoidance of breastfeeding [2]–[4]. The standard of care in lower income countries have been simplified, generally shorter, and less expensive regimens [5]–[11]. These regimens have included primarily single dose nevaripine (sdNVP) or short course regimens comprised of single or two drugs administered at the later stages of pregnancy [5]–[11]. The ultimate efficacy of these regimens maybe reduced in breastfeeding populations due to postnatal transmission [12]. Currently the World Health Organization (WHO) recommends a two-tiered approach for prevention of MTCT (PMTCT) in low income countries that includes provision of HAART for HIV-infected pregnant women in need of therapy for their own health to supplement the simplified regimens. However, the data on safety and effectiveness of HAART for PMTCT largely stems from experiences in higher income countries. There have been few reports that have assessed the impact of HAART in further reducing MTCT in high HIV seroprevalence and resource-limited settings [13].

Haiti has the highest prevalence of HIV (2.2%) of any nation outside of sub-Saharan Africa [14]. The *Groupe Haïtien d'Études du Sarcome de Kaposi et des Infections Opportunistes* (GHESKIO) located in Port-au-Prince is the largest voluntary counseling and testing center (VCT) for HIV in Haiti. In 1999, in collaboration with the Haitian Ministry of Health, GHESKIO established a program whose goals were to reduce the rate of MTCT and decrease mortality in infants born to HIV-infected mothers. The standard of care in Haiti for PMTCT was a shortened course of zidovudine during the latter stages of pregnancy (scZDV) for HIV infected pregnant women and for their infants from March 1999 until early 2003[8], [9]. With the availability of HAART in 2003, the program shifted to a two-tiered approach consistent with that recommended by WHO [11]. Pregnant women with advanced disease (as indicated by CD4 cell count and WHO stage of disease) were prescribed HAART and those who did not meet WHO eligibility criteria were given monotherapy with scZDV as per contemporary Haitian Ministry of Health guidelines. GHESKIO has published reports on its success with HAART therapy in both HIV infected adults and children in urban Haiti [15], [16].

Prior to the institution of PMTCT, 60% of the Haitian children with suspected HIV infection died before six months of age [17]. Other resource-poor settings have also reported a higher and earlier infant mortality in HIV-1 infected children than seen in the developed world [18]–[20]. Although highest in those infants who are HIV-infected, the excess infant mortality extends to all children born to HIV-infected mothers. At GHESKIO infant mortality rate was 200 per 1000 live births in the first 15 months of life at inception of the program. This rate was similar elsewhere in Haiti as MTCT programs were being initiated for example-230 per 1000 live births in a rural setting in Mirebalais [21].

In this study, we followed children born to HIV-1 infected women in the PMTCT program at GHESKIO for their first 15 months of life. The cohort encompassed infants born between 1999 and 2005. Our goals were to: 1) evaluate the program in the context of its effectiveness in reducing pediatric HIV infection and increasing survival of infants born to HIV-1 infected women 2) To identify risk factors for acquiring pediatric HIV and mortality in the first 15 months of life. Sequential introduction of interventions over a span of 6 years, gave us the opportunity to identify key elements of a successful PMTCT program as well as barriers to effective implementation. Although there is data on the effectiveness of specific interventions in controlled clinical trials and pilot projects [22], the present study is unique in being in the context of implementation of an evolving PMTCT program.

## Results

### Description of Cohort

Between 1999 and 2005, 551 infants were born to 508 HIV-infected mothers. There were nine twin deliveries in the cohort. Each twin was regarded as an individual birth in terms of outcome. Thirty-four mothers had more than one child during the period of study, 11 of whom had previously lost a child (and in one case twins) during the study. Twenty percent of the children had birth weights ≤2.5 kg. The majority of infants (85%) were formula-fed. Forty-five women breastfed and 36 women reported mixed feeding of breast milk and supplemental foods in the cohort.

Table 1 lists baseline characteristics of the entire cohort and compares those enrolled before and after the availability of HAART. Overall, median maternal age was 27 years (interquartile range [IQR] 23–32) and median maternal weight 61 kilograms (kg) (IQR 55–68) at time of enrollment. Nineteen percent of the women tested were positive for syphilis by rapid testing. Of the 101 women that were positive by rapid testing, 70 were confirmed by TPHA making the prevalence of syphilis in HIV-infected women 13%. Median Hgb levels were 9.0 g/dl (IQR 8–10) at study entry; well below the level recommended by the WHO for pregnant women (≥11.0 g/dl) [23]. The median CD4 count was 462 cells/mm^3^ (IQR 289–686). The clinical stage of disease was less advanced and there had been fewer prior deliveries in mothers in the post-HAART era suggesting that less symptomatic women were presenting for screening and care as the study progressed. However, their CD4 count on enrollment and time of presentation during the pregnancy did not change over the course of the study. Although the percent opting for formula did not change more infants were exclusively breastfed once HAART was introduced.

**Table 1:** Cohort comparison before and after the introduction of HAART prophylaxis.

Variable*	N+	Total	Pre-HAART	Post-HAART	p-Value#
***Maternal Demographic Characteristics***					
Age, Median (IQR), years	547	27.0 (23.0–32.0)	28 (23–33)	27 (23–31)	0.30^2^
Weight, Median (IQR), kg	512	60.8 (55.4–67.7)	62 (56–68)	60 (54–67)	0.15^2^
Parity, Mean	490	1.7	2.5	1.3	<0.001^2^
Gestation at entry, Mean, mths	547	5.4	5.5	5.3	0.34^2^
***Maternal Clinical Characteristics***					
CD4 count, Median (IQR), cell/ul	515	462.0 (289.5–686.0)	460 (314–730)	462 (284–664)	0.30^2^
Baseline Hgb levels, Median (IQR)	469	9 (8–10)	9 (8–10)	9 (8–10)	0.32^2^
Maternal stage of disease	526				<0.001^1^
Asymptomatic		335 (60)	103 (49.5)	232 (73)	
Symptomatic		58 (11)	20 (9.6)	38 (12)	
Clinical stage AIDS		133 (24)	85(41)	48 (15)	
Maternal Syphilis	543				0.40^1^
Positive		101 (19)	44 (21)	57 (17)	
Negative		442 (81)	172 (80)	270 (83)	
Maternal Death	508				0.21^1^
Alive		483 (95)	189 (93)	294 (96)	
Death		25 (5)	14 (7)	11 (4)	
***Infant Characteristics***					0.38^1^
HIV Status of Infant	467				
Negative		420 (90)	160 (88)	260 (91)	
Positive		47 (10)	21 (12)	26 (9)	
Gender	551				0.41^1^
Female		284(52)	116(54)	168(50)	
Male		267(48)	100(46)	167(50)	
Birth weight	491				0.34^1^
>2.5 Kg		395(80)	161(83)	234(79)	
≤2.5 kg		96(20)	34(17)	62(21)	
***Feeding***	551				<0.001^1^
Formula		469(85)	183(85)	286(86)	
Breast-fed		46(8.3)	9(4)	37(11)	
Mixed		36(6.5)	24(11)	12(4)	
***Infant death <15mths***	551	84(15)	46(21)	38(11)	0.002^1^

* Unless otherwise indicated, data are presented as number (%)

+ Number for whom data was available

# Difference between Pre-HAART and Post-HAART cohort using: ^1^ Pearson's chi-square test;^ 2^ Wilcoxon Test

### Impact of PMTCT in reducing transmission of HIV-1

Overall HIV infection in infants with sufficient follow-up to establish a laboratory diagnosis of HIV-status (467 of 551) was 10% (47/467; 95% CI 7.5–13.2). This represents a three-fold reduction from historically documented levels of 27% [17]. In 22 of the 47 HIV-positive infants the timing of infection could be determined. Of these 12 were infected *in utero* (positive test within 72 hours of birth) and 10 were infected peripartum (negative test at birth but a subsequent positive test). Eighty-four infants had an undetermined HIV-status due to early death and loss to follow-up.

**Figure 1:**
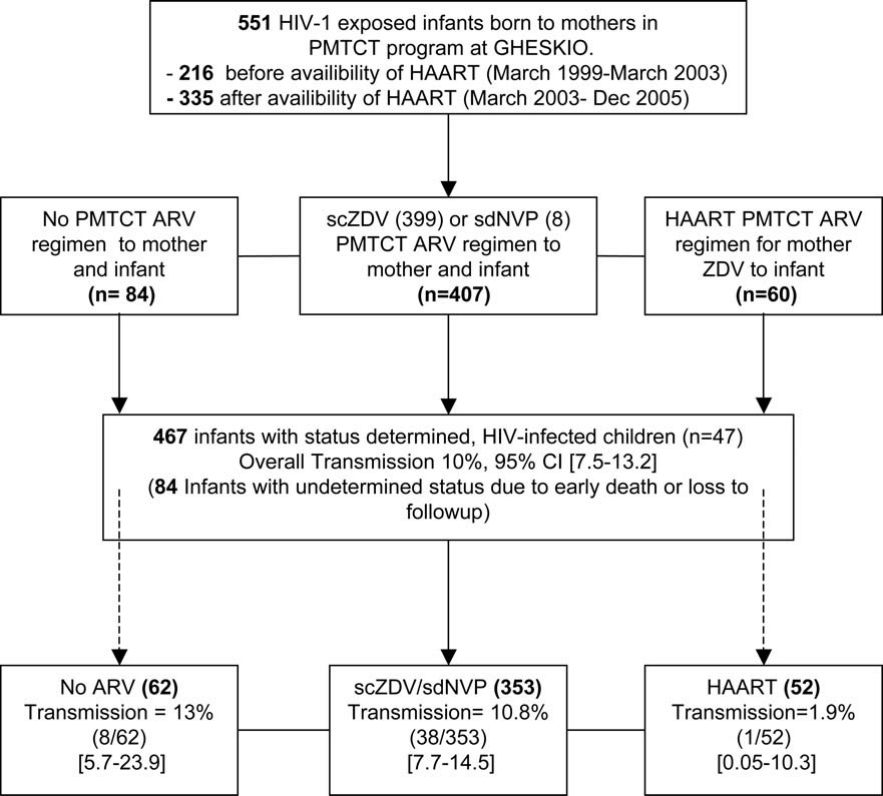
Profile of Cohort. Population used to estimate rate of mother-to-child transmission of HIV in the GHESKIO PMTCT program in Port-au-Prince, Haiti between March 1999 through December 2005. Cohort is divided based on the date of initiation of HAART for both individual health and PMTCT. Among the 47 HIV-infected infants, 38 had received scZDV alone, eight had received no-prophylaxis and 1 had received HAART.

We compared the transmission rates by ARV regimen used for prophylaxis. Among the 407 women receiving monovalent ART for prophylaxis the median duration of therapy was 32 days (IQR 21–46). The great majority (98%) received scZDV. Only eight received sdNVP. The overall transmission rate with monovalent therapy was 10.8% (38/353 infants with determined status, Figure 1). Sixty pregnant women received HAART for a median duration of 61 days (IQR 25–140). Of the 52 infants with final status available only one infant was HIV-1 infected (1.9%, Figure 1).

### Risk factors for MTCT of HIV-1

Univariate analysis on a selected set of parameters showed low maternal CD4 count, low maternal Hgb, low birth weight, and female gender to be associated with HIV-1 transmission (Table 2). Significantly fewer infants were infected among those who had received complete MTCT therapy (as defined by both maternal and infant treatment) (Table 2). In multivariate analysis based on the 421 pairs on which complete data was available only low birth- weight remained significantly associated with transmission (OR 4.79, 95% CI 2.15–10.67, p = 0.0001) when controlling for all other variables. Relatively few, 81, received breast milk limiting the power of analyzing the role of feeding choice as risk factor for transmission.

**Table 2:** Risk factors associated with **transmission** of HIV-1 from mother to infant.

Variable*	N	Negative n = 420	Indeterminate n = 84	Positive n = 47	p -Value
***Prophylaxis (Mother+Infant)*** a					**0.016** 1
None	84	54 (64)	22 (26)	8 (9.5)	
Incomplete	194	145 (74)	31 (16)	18 (9.3)	
Complete	273	221 (81)	31 (11)	21 (7.7)	
***Stage of Maternal Disease***					0.067^1^
Asymptomatic	335	264 (79)	48 (14)	23 (7)	
Symptomatic	58	42 (72)	13 (22)	3 (5)	
Aids	133	93 (70)	22 (16)	18 (13)	
Missing	25	21 (84)	1 (4)	3 (1.2)	
***Maternal Syphilis***					0.39^1^
RPR Positive	101	71 (70)	21 (21)	9 (9)	
RPR Negative	442	344 (78)	61 (14)	37 (8)	
Missing	8	5 (62)	2 (25)	1 (20)	
***Maternal CD4 counts^+^***	515	480 (322–745)	404 (243–585)	409 (284–578)	**0.004** 2
***Maternal Hgb values^+^***	469	9.2 (8.5–10.1)	9.0 (7.7–10.0)	9.0 (8.0–10.0)	**0.002** 2
***Birth weight***					**<0.001** 1
>2.5 kg	395	325 (82)	47 (12)	23 (6)	
≤2.5 kg	96	58 (60)	22 (33)	16 (17)	
***Gender***					**0.02** 1
Female	284	203 (71)	50 (18)	31 (11)	
Male	267	217 (81)	34 (13)	16 (6)	

* Continuous variables (^+^) represented as median value (IQR), all others as frequencies (%, calculated by row.

a Sixty received HAART, eight received sd-NEV, rest received monotherapy with AZT.

1 Pearson's chi-square test; ^2^ Kruskal-Wallis test

### Impact of PMTCT on Infant Mortality

**Figure 2:**
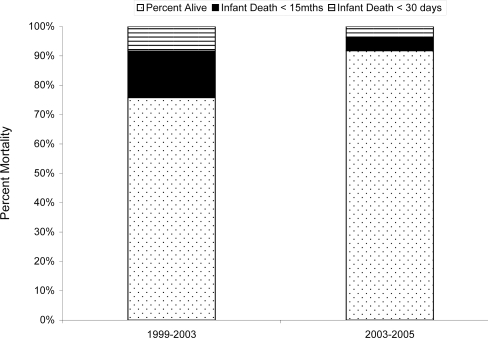
Mortality Pre and Post the availability of HAART. The overall reduction in mortality is significant and extends to both the neonatal and infant age ranges.

The overall mortality rate was 15.2 per 100 live births (95% C.I, 12.1–18.8). Twenty-three deaths (27% of the total deaths) occurred in the first 30 days of life. The mortality rate in children prior to HAART availability was 0.23 per year followed [0.16–0.30], and after HAART fell to 0.12 [0.08–0.16], p<0.0001. Survival improved in both the first 30 days of life and subsequently up to the 15 month end of follow-up (Figure 2). In the multivariate model, the availability of HAART was significant after multiple imputations (p = 0.032), (OR, 2.03; [1.06–3.87]), suggesting that HAART and interventions introduced since 2003 have contributed to a continued decrease in child mortality seen in both the 2004 and 2005 birth cohorts. Women with live births before HAART did not differ significantly from women delivering after HAART in demographics, maternal Hgb level, CD4 counts or frequency of low-birth weight infants although they had less advanced clinical disease and had had fewer prior births (Table 1).

### Risk factors for Infant and Neonatal Mortality amongst HIV exposed infants

A number of factors were predictive of infant mortality by univariate analysis including infant HIV-1 infection, low birth weight, maternal syphilis, anemia, low CD4 count, clinical stage of maternal disease and whether mother was living at 15 months (Table 3). Completion of PMTCT therapy also associated with decreased infant mortality (p = 0.021). When comparing neonatal to post-neonatal (31 days–15 months) mortality, the risk factors that were significantly associated with neonatal mortality were infant's HIV-status and maternal syphilis (Table 4). The full contribution of HIV status to neonatal mortality was hard to determine as many deaths occurred before a confirmatory PCR assay could be performed (Table 4). A higher neonatal mortality in those whose mother had syphilis remains unexplained, particularly in view of documented single-dose maternal penicillin treatment.

**Table 3:** Risk factors associated with infant **mortality**

Variable*	N	Infant Alive n = 467	Infant Died n = 84	p-Value^1^
***Prophylaxis (Mother+Infant)***				**0.026**
None	84	63(75)	21(25)	
Incomplete	194	168(87)	26(13)	
Complete	273	236(86)	37(14)	
***Maternal Status***				**0.021**
Alive	521	446(86)	75(14)	
Dead	30	21(70)	9(30)	
***Stage of Maternal Disease***				**<0.001**
Asymptomatic	335	300(90)	35(10)	
Symptomatic	58	47(81)	11(19)	
Aids	133	98(74)	35(26)	
***Maternal Syphilis***				**0.008**
RPR Positive	101	76(75)	25(25)	
RPR Negative	442	385(87)	57(13)	
Unknown	8	6(75)	2(25)	
***Maternal CD4 counts*** **^+^**	515	474(302–710)	384(242–552)	**<0.001** 2
***Maternal Hgb values*** **^+^**	469	9.4(8.4–10.1)	9(8–10)	**<0.001** 2
***Infant HIV Status***				**<0.001**
Negative	420	395(94)	25(6)	
Indeterminate	84	43(51)	41(49)	
Positive	47	29(62)	18(38)	
***Gender***				**0.021**
Female	284	231(81)	53(19)	
Male	267	236(88)	31(12)	
***Birth weight***				**<0.001**
>2.5 kg	395	357(90)	38(10)	
≤2.5 kg	96	56(58)	40(42)	

* Continuous variables (^+^) represented as median value (lower quartile-upper quartile), all others as frequencies (%, by row).

1 Pearson's chi-square test unless otherwise indicated; ^2^ Wilcoxon Test

**Table 4:** Univariate analysis comparing early and late infant **mortality**

Variable*	N	Neonatal mortality (≤30 days) n = 23	Post-neonatal mortality (31–450 days) n = 61	p-Value^1^
***Prophylaxis (Mother+ Infant)***				0.16
None	84	4(5.0)	17(20)	
Incomplete	194	5(2.6)	21(11)	
Complete	273	14(5.1)	23(8.4)	
***Maternal Status***				0.71
Alive	521	21(4.0)	54(10)	
Dead	30	2(7.0)	7(2.3)	
***Stage of Maternal Disease***				0.51
Asymptomatic	335	8(2.4)	27(8.0)	
Symptomatic	58	3(5.2)	8(14)	
Aids	133	12(9.0)	23(17)	
***Maternal Syphilis***				**0.04**
RPR Positive	101	11(11)	14(14)	
RPR Negative	442	12(3)	45(10)	
***Maternal CD4 counts*** **^+^**	79	480(230–552)	372(249–541)	0.9^2^
***Maternal Hgb values*** **^+^**	77	8(8–10)	9(8–10)	0.72^2^
***Infant HIV status***				**0.048**
Negative	420	7(2.0)	18(4.3)	
Indeterminate	84	15(18)	26(31)	
Positive	47	1(2.0)	17(36)	
***Gender***				0.8
Female	284	14(5.0)	39(14)	
Male	267	9(3.3)	22(8.2)	
***Birth weight***				0.29
>2.5 kg	395	11(2.8)	27(7.0)	
≤2.5 kg	96	12(12.5)	28(29)	

* Continuous variables (^+^) represented as median value (lower quartile-upper quartile), all others as frequencies (%, by row).

1 Pearson's chi-square test unless otherwise indicated; ^2^ Wilcoxon Test

In multivariate analysis performed on 421 with complete data sets, low-birth weight (Odds Ratio OR, 7.08; [95% CI 3.43–14.58, P<0.0001] and HIV-status of the infant (OR, 7.74; [2.99–20.03], P<0.0001) were the only risk factors that remained significantly associated with infant mortality. After multiple imputations to account for missing data, maternal stage of disease and maternal syphilis emerged as additional risk factors.

In our cohort, we found a significant correlation between low maternal Hgb and low CD4 counts (p<0.001) and HIV positive status of mother (P<0.01). The effect of an interaction between the variables was however not statistically significant and did not influence the ability of the overall model including all factors to predict mortality with a p<0.0001.

## Discussion

We report success in using both single drug short course regimens and a two-tiered approach in which HAART is prescribed for women with advanced disease as per WHO criteria and single drug regimens are given to all others. Mothers enrolled between 1999–2003 received only scZDV or sdNVP. In 2003, HAART became available in Haiti and mothers enrolled from March, 2003 until the end of the current analysis (infants born through December, 2005) received either scZDV, sdNVP or HAART as indicated by CD4 cell count and clinical staging. To date there have been few reports on the safety and effectiveness of HAART in HIV-infected pregnant women in low-resource countries though such a 2-tiered program has been suggested as a means to gain maximum impact of PMTCT [13], [24].

### Impact of PMTCT in reducing transmission of HIV-1

The reduction in transmission is encouraging considering that 35% of the women received less than the recommended duration of therapy and 15% no prophylaxis at all. Furthermore, these estimates may represent upper limits as adherence to therapy was not assessed pharmacologically and self-reporting has been shown to be subject to bias [25], [26].

Although reduced from historical controls transmission rate in the cohort of women given exclusively single drug prophylaxis versus those that were part of the two-tiered program (either ZDV or HAART if indicated) was similar (12% versus 9.1%). However, in 60 women who received HAART transmission rate was 1.9%, similar to that seen in developed countries suggesting that prophylaxis with HAART alone where possible would be more effective than monotherapy. No comparison of HAART with dual therapy or more prolonged monovalent therapy can be inferred from this trial and in that respect emphasizes the need to look comprehensively at the optimal approach to PMTCT in the developing country setting.

### Impact of PMTCT on Infant Mortality

Children born to HIV-1 infected mothers are at increased risk of mortality [19]. Infant mortality prior to the introduction of antiretrovirals for prevention of MTCT was 23 per 100 live births [17] and it continued at that rate through the first three years of our study. Mortality decreased dramatically about one-half way through the period coincident with the introduction of HAART and was seven per 100 in the year 2005. Although more women having clinical AIDS in the pre-HAART cohort could have contributed to the worse prognosis of the infants, in multivariate analysis reduced mortality in infants born post-HAART remained significant even when adjusted for maternal stage of disease.

As has been demonstrated in earlier studies, low birth weight and infant HIV-status emerged as significant risk factors for infant mortality [27], [28]. A recent study from West Africa (MTCT-Plus Initiative) observed a higher proportion of low birth weight in neonates receiving HAART versus simplified regimens [13], [29] raising the possibility that HAART may have contributed to the low birth weight. We did not observe an increased number of low birth weight infants born to women receiving HAART for prophylaxis. A high percentage of the infant mortality occurred in the first month of life. Surprisingly the neonatal mortality was not disproportionately due to low birth weight.

The difference in mortality rate before and after HAART introduction (p<0.0001) and the reduction in HIV MTCT though impressive occurred in the face of a changing population presenting to GHESKIO and was only a part of what has become an integrated and comprehensive maternal and child care programs. For example, beginning 2001, co-trimoxazole was introduced for all HIV-exposed infants in the PMTCT program beyond 6 weeks of age. Co-trimoxazole prophylaxis has proven to be a cost-effective intervention with demonstrated success in increasing infant survival in both African settings [30], [31] and at GHESKIO [32]. Vaccination with pneumococcal vaccine and conjugated H. influenzae was also begun in 2001. Both of these interventions preceded the observed decline in mortality in 2004 and 2005.

### Role of Feeding in Transmission and Mortality

There is a consensus emerging that exclusive breastfeeding is the most viable option in resource-limited settings for survival of an HIV-exposed infant [33], [34]. While infant feeding practice was not associated with a change in HIV-1 transmission or neo-natal and post neonatal mortality (data not shown) we were severely limited in power to make any meaningful conclusions as 85% of women chose to formula-feed. Regardless of mode of feeding, complementary food introduced to infants beyond six months of age must be of adequate caloric and nutritional value to ensure continued growth. Recognizing that growth faltering occurs after weaning from formula or breastfeeding, a nutritional intervention study is underway at GHESKIO.

### Lessons learned and new directions for the future

In addition to the factors identified by multivariate analysis, low birth weight and infant HIV status, the variables identified as significant by univariate analysis deserve consideration when developing models for monitoring progress and program improvement. For instance maternal syphilis and anemia are areas that with remedial action could positively impact the effectiveness of the program. The high percentage of HIV-infected pregnant women with syphilis certainly reinforces the need to integrate testing for both syphilis and HIV to all women.

Our study was limited in that it was not a randomized intervention study. Anticipating the rapid evolution of PMTCT practice during this period we employed components of a continuous quality improvement model in a programmatic setting. As a result, we were unable to dissect the effects of individual interventions (such has HAART for mothers versus co-trimoxazole for infants). Additionally, only 421 of mother-infant pairs (out of 551) have non-missing data for all risk factors reducing the power of the multivariate analysis. Finally the unknown HIV infection status of the 84 infants who died or were otherwise lost to follow-up may have altered the transmission frequency and risk factors in ways that we cannot predict. Never-the-less the 7.2% lost to follow-up rate (after exclusion of the 42 early deaths) seems low in a country with the high level of social disruption currently seen in Haiti.

This study demonstrates how rigorously analyzed data in PMTCT programs can be used to make evidence-based decisions. With risk factors better defined by this and other papers a prospective, comprehensive examination of risk factors for pre-, peri- and postnatal HIV transmission and improvement of infant survival could have a major impact on one of our most successful interventions to reduce the impact of HIV.

In conclusion, we analyzed the data collected over six years in a PMTCT program established in resource-limited Haiti to answer whether: 1) the current practices within the PMTCT program are effective in reducing transmission and mortality and 2) we can identify risk factors for transmission and mortality that will guide future interventions. We find in the Haitian setting that with the introduction of HAART can limit transmission to levels that approach those in industrialized countries. However, early infant mortality continues to be observed in HIV-1 exposed children suggesting that factors such as maternal anemia, prevention of low birth weight infants are important areas of investigation. Challenges remain in enhancing coverage and devising mechanisms to assure early presentation in pregnancy and compliance. Finally, we have demonstrated the ability of PMTCT programs to adopt complicated anti-retroviral regimens with success despite limited human resources and anticipate with expanded coverage of HAART in a comprehensive maternal-infant program continued gains in coming years.

## Methods

### PMTCT Model and Clinical Care of Participants

The GHESKIO Centers located in Port-au-Prince, Haiti provide VCT services for over 26,000 individuals yearly and care for the approximately 15% of those who are HIV infected. The comprehensive program integrates VCT with reproductive health (family planning), treatment for sexually transmitted disease, diagnosis and therapy of tuberculosis and pre- and post-natal care for HIV-infected mothers and their infants. The GHESKIO model has been previously described [35]. The PMTCT services provided within the comprehensive program serve as a laboratory for the Ministry of Health to inform national recommendations for HIV care.

The participants in this study included all available HIV-infected pregnant women and their live-born infants enrolled between March 1999 and December 2005 and followed until 15 months of age and/or a diagnosis of pediatric infection with HIV could be made. All HIV-infected pregnant women were individually counseled, informed of their HIV status, and referred to the antenatal clinic where a series of regular prenatal visits with the obstetrical team were scheduled. An agreement was signed by the women to participate in the provision of services at GHESKIO and to allow the physicians to review their records. The activities were reviewed by the GHESKIO IRB and deemed to be provision of services.

#### Antenatal Care and Antiretroviral Regimens

At the onset of the pilot program (March 1999) scZDV was made available to every HIV-infected pregnant woman with a hemoglobin (Hgb) level >8.5 g/dl before or at an estimated 36 weeks of gestation (300 mg twice daily) and during labor (300 mg every 3 hours until delivery)[8], [36], [37]. Women who presented for care close to time of delivery or had Hgb below 8.5 g/dl were instructed to self-administer a single dose (sd) of 200 mg of NVP at the onset of labor. Beginning in March 2003, pregnant women with clinical indications or with a CD4 count of <350 cells/dL were administered zidovudine/lamivudine/nevirapine (ZDV/3TC/NVP) or, if the mother was anemic, stavudine/lamivudine/nevirapine (d4T/3TC/NVP). Treatment was started for maternal health, and continued through pregnancy and after delivery. Pregnant women not eligible for HAART continued to receive prophylaxis that included either scZDV or sdNVP. All infants were started on ZDV at 2 mg per kilogram every six hours for seven days irrespective of maternal drug regimen. Compliance to therapy was monitored through self-reporting, pill counts and pharmacy records. Prenatally, mothers were counseled on infant feeding options. They were given the choice between formula, (provided at no cost for the first nine months) and breastfeeding. Women who chose not to breastfeed were instructed on preparation of formula, safe sources of water, and oral rehydration.

Virtually all deliveries occurred vaginally in a home setting accompanied by a traditional birth attendant [38]. Mothers or other family members were encouraged to bring infants to the clinic within three days of life at which point infants were started on ZDV at 2 mgm/kg every six hours for seven days.

#### Follow-up

Visits were scheduled for mother and infant twice a month for six months and then once a month until 24 months of age. Infants were provided trimethoprim-sulfamethozaxole (co-trimoxazole) (150 mg/m^2^/24 hours for three consecutive days per week) beginning at six weeks of life following recommendations [32], [39]. Since 2003, infants diagnosed with HIV infection have been started on HAART based on WHO criteria [16]. Routine immunizations in the Expanded Program on Immunization for Haiti were provided as well as unconjugated pneumococcal and conjugated haemophilus influenzae vaccines from 2001.

At each visit, the infant was weighed and sufficient formula given to provide nutrition until the next visit. Permission was requested to make home visits (one field worker assigned for 50 women) if the women did not return for their recommended visits. Children were considered lost to follow-up if they did not visit for a period of three months and could not be located by the field worker.

### Laboratory

#### Maternal Testing

Rapid whole blood HIV antibody testing was done using Determine HIV 1/2 (Abbott Laboratories, Abbot Park IL) and Capillus HIV 1 and 2. (Trinity Biotech, Bray, Ireland) [40], [41]. The diagnosis of HIV infection was dependent on both results being positive. If the results of the results were discrepant, the laboratory performed a Western Blot for final designation of HIV status. Routine syphilis was done using Rapid Plasma Reagin (RPR; Becton Dickinson, Sparks, MD) with confirmation by FTA-ABS. Women with syphilis were treated and their infants treated as indicated based on the completeness of maternal therapy. CD4 cell counts are determined by flow cytometry using FACSCount (Becton Dickinson Biosciences, San Jose, CA). Hgb values were determined using a Coulter counter (Beckman Coulter Inc, Miami, FL). In some cases data entry was by integers and the complete value to the first decimal point could not be retrieved.

#### Infant HIV status

HIV infection in children born of HIV-infected mothers was determined by a nucleic acid sequence-based assay, NASBA (bioMerieux Boxtel, The Netherlands) for the first 3 years of the study. Since that time, polymerase chain reaction (PCR) testing for HIV-1 RNA (COBAS AMPLICOR, Roche Molecular Diagnostics, Branchburg, NJ) has been performed on HIV-exposed infants at birth, 2–3 months and six months of age. Both have cut-off values of 400 copies/ml. In 2003, a simplified p24 antigen assay was introduced for early diagnosis of infant HIV-1 infection [42]. HIV antibody test (ELISA) was repeated every three months until seroreversion confirmed that maternal antibodies had disappeared. Criteria for determining infant HIV status were based on serology and nucleic acid detection as previously described [32]. Clinical criteria did not enter into the determination of HIV status.

### Definitions and Measurement of Risk Factor Variables

Maternal CD4 counts and Hgb values were examined from enrollment, during pregnancy and within three months after delivery. For this analysis, we used the CD4 measurement closest to the date of delivery and the earliest Hgb obtained in the course of the pregnancy.

Women within our cohort received one of three regimens: scZDV, sdNVP or HAART. Complete maternal treatment was defined as receiving 28 days of scZDV antepartum or sdNVP at the onset of labor. As HAART regimens included NVP, women receiving less than 28 days of HAART before delivery were considered to have completed their prophylaxis. Infant treatment was complete if the infant received one week of ZDV started within 72 hours of birth. Prophylaxis for PMTCT was considered complete only if both the mother and infant received the recommended duration of therapy, based on a combination of pharmacy records and self-reporting by the women.

Maternal and infant deaths were defined as deaths occurring within 15 months after delivery. Because infant deaths in the first month of life are often attributable to perinatal events these were separately analyzed. Birth weight of the infant was deemed to be the first available measurement within 14 days from birth as weights do not generally change dramatically from birth weight during that time.

An infant with a positive PCR within three days of life was classified as infected in utero. An infant with a negative PCR at birth but subsequently confirmed to be HIV positive was considered to have been infected in the peripartum or potentially through breast milk if that had been offered. All others who were HIV positive but not assignable to in utero or peripartum infection because of lack of early PCR were scored as infected but with uncertainty as to timing and route of infection [43].

To capture the effectiveness of transition from limited to more complete prophylactic regimens and interventions, we divided the cohort based on date of initiation of HAART in March of 2003. All women prior to that date received only scZDV or sdNVP. Women in the later portion of the cohort received either HAART, scZDV or sdNVP based on CD4 counts and clinical stage.

### Statistical Analysis

Analysis of all data presented in this manuscript was handled with patients being de-identified. All clinical, demographic and laboratory data on mothers and their infants were incorporated into an integrated data management system. Univariate analysis, to assess covariates as predictors of transmission and infant mortality were performed using Chi-square test for category variables, and Wilcoxon rank-sum/Kruskal-Wallis test for continuous variables.

Multivariable logistic regression models were performed on mother-infant pairs that had complete data for all risk factors included. One model, utilized HIV-status of the infant as the outcome, the other utilized infant mortality. Risk factors included in the models were: HIV status of infant, low birth weight, maternal death within 15 months from infant birth, treatment status, maternal Hgb, maternal clinical stage of disease, maternal syphilis, breast milk exposure and whether enrolled before first HAART date or after. In a separate analysis missing values for risk factors in the multiple regression models were imputed using the flexible semiparametric algorithm in Hmisc package in R [44] (http://cran.rproject.org/web/packages/Hmisc/index.html).

Interaction effects between birth weight and other risk factors were tested with a single global (pooled) test and were not significant. Therefore, interaction terms were not included in the models.

Mortality rate per year of follow-up was defined as the number of deaths divided by the follow-up time (censored at 15 months), and 95 percent confidence intervals were calculated with the use of Poisson distribution. Comparison of rates was based on normal-theory test. Proportions of transmission by treatment status were reported and the 95 percent confidence intervals were computed with the use of the binomial distribution. All calculations were done using R version 2.6 (software available at www.r-project.org).
